# Ending the pandemic is not a matter of chance; it’s a matter of choice

**DOI:** 10.2471/BLT.22.287849

**Published:** 2022-02-01

**Authors:** Viroj Tangcharoensathien, Tedros Adhanom Ghebreyesus

**Affiliations:** a International Health Policy Program, Ministry of Public Health, Tiwanon Road, Nonthaburi 11000, Thailand.; b World Health Organization, Geneva, Switzerland.

Two years since its emergence, the coronavirus disease 2019 (COVID-19) pandemic continues to take a heavy toll on economies, employment and livelihoods. The World Bank predicts a strong but uneven economic recovery in 2022; however, economic growth is concentrated in a few major economies. While most high-income countries are predicted to regain their pre-pandemic per capita income by 2022, only one third of emerging market and developing economies will.^1^ Low-income countries are being left behind as the pandemic has reversed gains in poverty reduction, compounded by food insecurity and other long-standing challenges. 

The pandemic has demonstrated the importance of investments in public health, but the sluggish economic recovery in developing countries reduces the capacity of governments to make those investments. Rebuilding health systems, progressing towards universal health coverage and strengthening public health functions require significant public resources. In the context of limited fiscal capacity and demand for adequate health budgets, political will and commitment to advance the health of people should prevail.

We advocate for governments to safeguard the health of their populations by sustaining and increasing budget allocation to match the need for building back stronger and more resilient health systems. In response to the 2008 financial crisis, several European countries implemented different levels of austerity policies, including cutting budgets for public sector programmes such as health, education and social welfare. A study in 15 European countries showed that countries where intermediate- and high-austerity measures were implemented had higher excess annual mortality than countries in the low-austerity group.^2^

As the pandemic has had a higher negative effect on the most vulnerable populations, calls exist to not only build back better, but fairer. Doing so requires strong and continued political leadership across different governments to redress entrenched and structural social inequality.^3^

Nowhere have those inequalities been demonstrated more clearly than in access to vaccines against COVID-19. The development of several safe and effective vaccines with World Health Organization (WHO) Emergency Use Listing in less than a year is a remarkable scientific achievement, but successful protection of populations relies on equitable access to vaccines in all countries.^4^ An estimated 72% of total global doses have been administered in upper-middle- and high-income countries, while only 1% of doses have been administered in low-income countries.^5^

The pandemic has created additional inequity, as affluent countries experience economic recovery while poorer countries face acute shortage of vaccines and therapeutics.^6^^,^^7^

Vaccine inequity is a moral failure and an economic and epidemiological failure. The pandemic will be prolonged unless this disparity is overcome. The emergence of other severe acute respiratory syndrome coronavirus 2 variants could threaten the uneven progress made so far.^8^

Ending the pandemic is not a matter of chance, it’s a matter of choice, and the choice is in our hands. WHO is therefore calling for a global effort to vaccinate 70% of the population of every country by mid-2022. Achieving this target will help to end the acute phase of the pandemic and will enable countries to reopen.

We call on manufacturers and countries that have already reached 70% vaccination coverage to prioritize COVAX Facility and the African Vaccine Acquisition Trust,^9^ and to work together to support those who are furthest behind.

Removing barriers to scaling up vaccine production capacity in low- and middle-income countries is needed, through technology transfer and by supporting a waiver of intellectual property rights, as most COVID-19 vaccine research has been funded by public resources.^10^

The pandemic will not end unless we end vaccine inequity, but vaccines alone will not achieve this goal. Consistent and tailored implementation of public health and social measures is needed, including surveillance, testing and sequencing of potential variants of concern. Individual risk mitigation measures including masks, hand hygiene, physical distancing and ventilation should continue to be implemented.

This theme issue of the *Bulletin of the World Health Organization*, launched at the Prince Mahidol Award Conference in January 2022, provides a collection of evidence and case studies on how countries are responding to the pandemic, including the use of data, the role of community health workers, how to build resilient supply chains, lessons learnt in sustaining essential health services for immunization, child nutrition, eye care, tuberculosis, maternal and child health, peritoneal dialysis and more. This issue offers valuable insights and lessons from which all countries can learn, for this pandemic and for preventing and mitigating the impact of future health crises.

Ending the acute phase of the COVID-19 pandemic must remain our immediate priority. But the true measure of success will be our ability to learn lessons from the pandemic and make the world safer for future generations.
